# A novel biomarker selection method combining graph neural network and gene relationships applied to microarray data

**DOI:** 10.1186/s12859-022-04848-y

**Published:** 2022-07-26

**Authors:** Weidong Xie, Wei Li, Shoujia Zhang, Linjie Wang, Jinzhu Yang, Dazhe Zhao

**Affiliations:** 1 School of Computer Science and Engineering, Northeastern University, Shenyang, China; 2 Key Laboratory of Intelligent Computing in Medical Image (MIIC), Northeastern University, Ministry of Education, Shenyang, China

**Keywords:** Graph neural networ, Feature selection, Biomarker, Spectral clustering

## Abstract

**Background:**

The discovery of critical biomarkers is significant for clinical diagnosis, drug research and development. Researchers usually obtain biomarkers from microarray data, which comes from the dimensional curse. Feature selection in machine learning is usually used to solve this problem. However, most methods do not fully consider feature dependence, especially the real pathway relationship of genes.

**Results:**

Experimental results show that the proposed method is superior to classical algorithms and advanced methods in feature number and accuracy, and the selected features have more significance.

**Method:**

This paper proposes a feature selection method based on a graph neural network. The proposed method uses the actual dependencies between features and the Pearson correlation coefficient to construct graph-structured data. The information dissemination and aggregation operations based on graph neural network are applied to fuse node information on graph structured data. The redundant features are clustered by the spectral clustering method. Then, the feature ranking aggregation model using eight feature evaluation methods acts on each clustering sub-cluster for different feature selection.

**Conclusion:**

The proposed method can effectively remove redundant features. The algorithm’s output has high stability and classification accuracy, which can potentially select potential biomarkers.

## Background

With the development and maturity of microarray technology, researchers can obtain a large number of gene expression values at once by DNA microarray technology, and these data can be used to analyze critical genes for disease diagnosis, drug development, and other tasks [1]. The difficulty of microarray data analysis is the large feature dimensionality and small sample size. Machine learning based feature selection methods can be used to select essential features from high dimensional data to solve this problem.

In the feature selection task, the purpose is to find a set of feature subsets of original features, which are highly redundant with the original features and significantly correlate with the label information. Feature selection is different from feature extraction, which obtains a set of representation information of low-dimensional space from high-dimensional space. Feature extraction can not explain the meaning of the representation of low-dimensional space and can not be well connected with downstream tasks [2]. Traditional feature selection tasks can be divided into filter, wrapper, and embedded methods.

The filter method does not rely on the machine learning model and solves the best feature ranking through the statistical calculation mode. It has high speed but low accuracy. The common filter methods mainly contain t-test [3], chi-squared test [4], and maximum information efficiency (MIC) [5], fisher score [6]. The wrapper method relies on a specific feature evaluator or machine learning model. It constantly looks for the best feature combination through the heuristic search algorithm. According to the return value of the evaluator as the fitness function, it can find the optimal feature subset under the feature classifier. However, local optimization and high time complexity are the disadvantages of the wrapper method. Common wrapper methods incorporates Stability Selection [7], Recursive Feature Elimination (RFE) [8], Genetic Algorithm (GA) [9] ,Artificial Bee Colony (ABC) [10], Ant Colony Optimization (ACO) [11] and Particle Swarm Optimization (PSO) [12]. The embedded method skillfully combines the feature selection process with the machine learning model, and outputs the feature subset through the weight parameters of the model. The effect of this method depends on the machine learning model, and not all models support the output of weight parameters. The common embedded method comprises Decision Tree(DT) [13], Random Forest Algorithm(RF) [14], and Linear Regression(LR) [15].

The hybrid feature selection algorithm combines the advantages of the above three algorithms and is the mainstream algorithm for feature selection tasks [16–18]. For example, researchers can combine the filtering method and packaging method to realize the rapid filtering of invalid features in the filtering method and reduce the time complexity of the packaging method to design an efficient packaging method for further selection and optimization of features. These methods have been widely used and reported and have achieved excellent results on mainstream microarray data sets. Salem et al. proposed a feature selection method, which combines genetic algorithm and information gain for feature selection to achieve high classification accuracy [19]. Jain et al. [20] proposed a two-stage hybrid feature selection method, which first uses the correlation based method to filter redundant features, and then uses the improved binary particle swarm optimization algorithm for further feature selection. Moradi et al. [21] proposed a hybrid feature selection method for microarray data classification, which combines local search strategy with particle swarm optimization algorithm to select feature subsets with low redundancy.

However, most current hybrid feature selection methods assume that the samples are independent and identically distributed or infer the relationship between the samples based on the data model. DNA microarray data is different from common natural data. Its biggest feature is that features (genes) are not independent of each other, but have rich dependencies. These relationships have been reported by a large number of literatures and have been sorted out in GeneMANIA. A large number of reported dependencies between genes, such as gene pathway, physical interaction, and other information. However, this prior knowledge information is ignored by most algorithms [22]. Existing studies have emphasized and demonstrated the importance of taking feature interactions into account for feature selection tasks. For example, probabilistic graphical model-based methods use information entropy and conditional probability to infer interactions between features, while interactions between genes do not follow probability distributions. The actual existence of pathways and co-expression relationships are underutilized in these methods [23]. Although methods based on mutual information, maximum correlation, and minimum redundancy emphasize feature interaction, simple mathematical models cannot infer complex gene interaction relationships.

The graph model adopts the form of nodes and edges, which can well represent the interaction relationship between non-independent and identically distributed data and is well applied to non-Euclidean structure data. Mainstream platforms for analyzing gene or protein interactions, such as GeneMANIA and STRING, are represented by graph structure [22]. The research of [24] and [25] is devoted to finding the characteristic genes of microarray data. Based on the graph structure data, the regularization technology is used to realize the feature selection in the graph structure. However, these methods do not capture the high-order connectivity of graph structure data and do not apply the prior knowledge in the existing database. Graph has been mathematically applied to social science [26, 27], protein interaction network [28], knowledge graph [29], and other research fields [30]. Graph neural network makes each node have global information representation through information dissemination and aggregation between nodes and fully excavates the feature interaction relationship and high-order connectivity information. However, this method has not been applied to the microarray data feature selection task.

The task of microarray data analysis differs from that of other data analysis in that a large number of proven feature dependencies already exist in microarray data. To better exploit these relationships and to refine some unknown relationships, we consider a graph structure to model the data and use graph neural network techniques to predict the unknown relationships between features. In addition, considering that there may be a high degree of redundancy among the features, we used clustering techniques to cluster the features based on the graph structure. Finally, considering that a single feature evaluation method may not be able to comprehensively and effectively assess the feature importance, we consider applying multiple evaluation methods to assess these features in each subgraph and use ranking aggregation to generate a unified ranking list, with the ultimate goal of obtaining a subset of features with low redundancy, high robustness, and practical significance.

This paper proposes an innovative biomarker selection method for microarray data. Our previous research has shown that graph neural networks can be a good guide to biomarker selection [31]. In the proposed method, the graph structure is used to establish the interactive information between genes, and each node represents a feature. The numerical correlation of genes and the correlation existing in prior knowledge are considered the edges between nodes in the graph. The proposed method uses graph neural network technology to spread and aggregate the information of each node and predicts the possible feature interaction through connection prediction technology. Then, in order to delete redundancy features, spectral clustering technology is applied to the graph. Each clustering subgraph is regarded as a feature subset with high self redundancy and low external redundancy. Each feature subset is a candidate feature subset to select the final marker gene. In order to ensure the reliability of the results, we use eight different feature evaluators to evaluate the candidate feature subset, input the results into a reliable sorting fusion algorithm, and finally output the feature subset.

The main contributions of this study include the following contents.

1. A comprehensive framework for feature selection of microarray data is proposed, which selects a subset of features with low redundancy and high robustness in order to take full advantage of the already validated dependencies between features, employs graph neural networks for data modeling, uses clustering ideas to cluster similar features on the graph structure, and outputs the best features on each subcluster by fusing the results of multiple feature evaluation methods. To the best of our knowledge, this is the first time that graph neural networks and feature ranking fusion methods are combined.
2. An innovative proposal is made to mine and extend the dependencies between features using graph neural network techniques. In the proposed method, a priori knowledge is used to build graph structure data, and information propagation and aggregation ideas are used to make the nodes corresponding to each feature get the ability to characterize global information, and then link prediction techniques are used to mine possible dependencies between features, and these dependencies are used to further analyze and cluster redundant features.
3. While filtering redundant features using the clustering idea, the features of each sub-cluster are further ranked using eight common feature evaluation methods, and the best features corresponding to each sub-cluster are obtained by generating a uniform ranking list using the ranking aggregation idea, so that a subset of features with low redundancy and high robustness can be selected.

The rest of this paper is organized as follows: the The Results part shows the experimental results, the Method part briefly introduces the overall framework of the proposed algorithm and describes each module in detail. Finally the Discussion and Conclusion parts summarizes the full text.

## Results

This section describes the proposed feature selection processing flow, firstly, how to pre-process the data and the initial filtering of features using T-test, followed by our improved binary difference evolution algorithm flow. Finally we present the improvement strategies for the scaling factor and fitness function of the binary difference evolution algorithm. In our experiments, the number of clusters set is 4. The number of iterations of the algorithm is determined based on the threshold $\varepsilon$. In our experiments, we chose $\varepsilon$ to be 0.01. In addition, for the parameters related to the eight feature evaluation methods used, we used the default parameters provided in the sklearn package. The detailed parameters are shown in Table 1.

**Table 1:** Parameter setting in experiment

Parameter description	Parameter setting
Number of clusters	4
Iterations	According to threshold $\varepsilon$
Threshold $\varepsilon$	0.01
T-test reserved features	100
evaluation method parameters	Sklearn default

### Cluster quantitative analysis

In the experimental process, firstly, a T-test was performed on all features, 100 groups of features were retained, the gene relationship matrix is obtained from GeneMANIA, and the Pearson correlation coefficient is calculated. The graph structure is established using the 100 groups of features, and the feature selection is carried out according to the proposed method. The number of clusters in this section is set to be 1–50, respectively. After feature sorting and fusion, the feature subset is taken as the final feature selection result, SVM is taken as the classifier, and the average *Acc* and *Auc* of 10 fold cross-validation are taken as the final evaluation index.

**Fig. 1:**
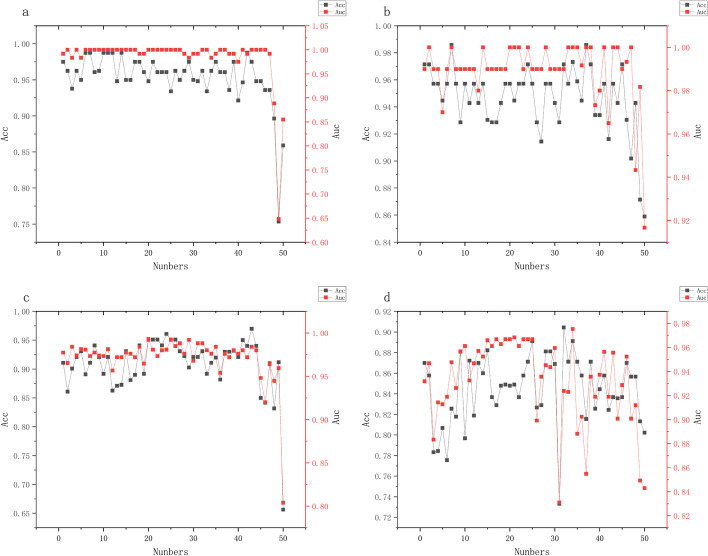
The relationship between the number of features (number of clusters) with *Acc* and *Auc*. **a** represents DLBCL data set, **b** represents leukemia data set, **c** represents prostate data set, and **d** represents ALL_4 data set

Figure 1 shows the relationship between the number of clusters with Acc and Auc on four different datasets, respectively. It can be found that as the number of clusters increases, redundant features are continuously introduced into the feature subset, resulting in a decrease in evaluation indicators. A smaller number of features (the number of clustered subclusters) can remove redundant features well, and when the number of features is very small, although a higher Acc index can be obtained, the Auc index may be lower, and the result stability is poor.

### Comparison with traditional algorithms

This section compares the proposed method with traditional machine learning feature selection methods, shown as Table 2, including linear regression model (liner), L1 regularization (lasso), random forest (RF), L2 regularization (ridge), feature recursive elimination (RFE) and decision tree (DT), It can be seen that the proposed method is superior to all classical machine learning algorithms when only one feature is adopted, which proves the superiority of the proposed method.

**Table 2:** The proposed method is compared with the classical method

Method	DLBCL	Leukemia	Prostate	ALL4
Liner (3)	0.820	0.943	0.842	0.849
Lasso (3)	0.909	0.936	0.921	0.830
RF (3)	0.923	0.915	0.932	0.806
Ridge (3)	0.820	0.943	0.842	0.849
RFE (3)	0.753	0.971	0.843	0.839
DT (3)	0.766	0.915	0.756	0.826
Ours (1)	0.975	0.971	0.911	0.870

The number of features used is indicated in parentheses

### Comparison with advanced methods

This section compares the proposed method with the advanced feature selection method, and the detailed results are shown in Table 3. Bold Fonts indicate the best results. It can be found from the table that the proposed method is still better than the advanced feature selection algorithm when the number of features is small. Unlike most hybrid methods, which require high time complexity, the proposed method only needs one aggregation calculation of graph neural network and a simple feature evaluation method to achieve efficient feature selection. In addition, considering the prior knowledge and feature dependence, the features selected by the proposed method have better interpretability and lower redundancy.

**Table 3:** Comparison between the proposed method and the advanced method

Datasets	Papers	Year	Features	Acc
DLBCL	Agarwalla et al. [32]	2018	15	0.900
DLBCL	Medjahed et al. [33]	2017	15	0.894
DLBCL	Wang et al. [34]	2017	15	0.809
DLBCL	Apolloni et al. [35]	2016	15	0.929
DLBCL	Wang et al. [36]	2015	15	0.936
DLBCL	Maulik et al. [37]	2013	15	0.918
DLBCL	Yu et al. [31]	2021	15	0.946
DLBCL	**Ours**	2022	**1**	**0.975**
Leukemia	Lu et al. [38]	2019	9	0.952
Leukemia	Sun et al. [39]	2018	3	0.927
Leukemia	Wang et al. [34]	2017	8.3	0.961
Leukemia	Tumuluru et al. [40]	2017	/	0.946
Leukemia	**Ours**	2022	**2**	**0.971**
Prostate	Samson et al. [41]	2021	3	0.830
Prostate	Khani et al. [42]	2020	5	0.922
Prostate	Musheer et al. [43]	2019	4	0.763
Prostate	Theera et al. [44]	2018	5	0.874
Prostate	Paredes et al. [45]	2017	24.32	0.928
Prostate	Gunavathi et al. [46]	2014	10	0.927
Prostate	**Ours**	2022	**5**	**0.931**

### Biomarker analysis

**Fig. 2:**
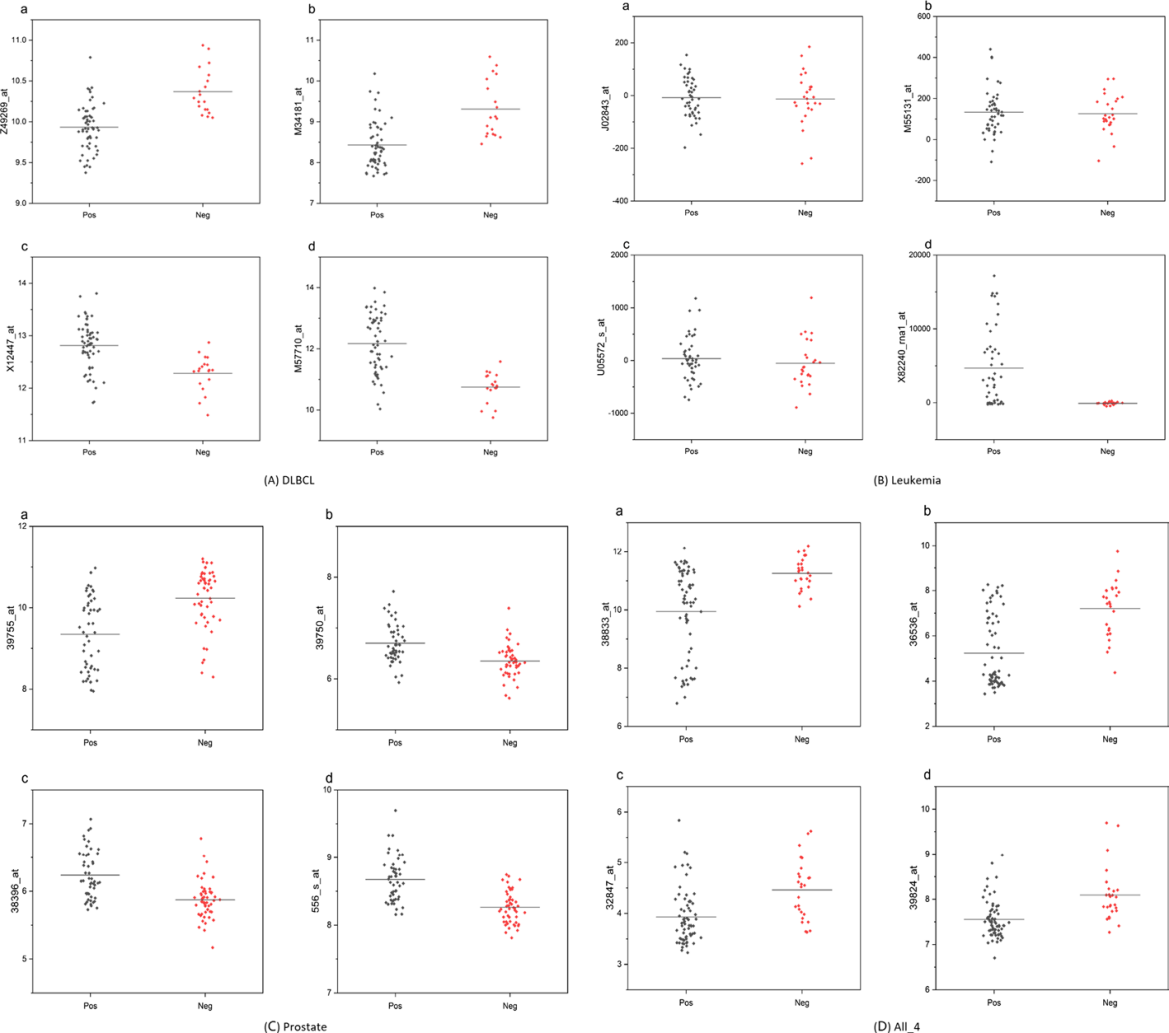
The distribution of biomarkers selected by the proposed method in positive and negative samples. **a** represents DLBCL data set, **b** represents leukemia data set, **c** represents prostate data set, and **d** represents ALL_4 data set

In this section, we further analyze the selected features of the proposed method. Figure 2a–d shows the results of the four data sets, respectively. The distribution of the four most essential probe ids selected by the proposed method in positive and negative samples is plotted in each data set. It can be seen that the features selected by the proposed method can effectively distinguish positive and negative samples, which have high diagnostic significance.

To further demonstrate the significance and validity of the selected biomarkers, we performed t-test analysis and heat map for the biomarkers selected by the proposed method on four datasets, where for each dataset we selected the best four biomarkers. the results of the t-test analysis are shown in Table 4. The table lists the probe IDs and significance levels corresponding to the selected biomarkers on the different datasets. Where * indicates significant at 0.05 level, ** indicates significant at 0.01 level, and *** indicates significant at 0.001 level. The results in Table 4 show that all the features selected by the proposed method are significant and 81.25% of the features have very high significance, which proves the validity of the features selected by the proposed method.

**Table 4:** The significance level of the features selected by the proposed method

Dataset	Probe ID	significant level	Dataset	Probe ID	significant level
DLBCL	Z49269_at	***	Leukemia	J02843_at	*
	M34181_at	***		M55131_at	*
	X12447_at	***		U05572_s_at	**
	M57710_at	***		X82240_rna1_at	***
Prostate	39755_at	***	All_4	38833_at	***
	39750_at	***		36536_at	***
	38396_at	***		32847_at	***
	556_s_at	***		39824_at	***

* indicates significant at 0.05 level, ** indicates significant at 0.01 level, and *** indicates significant at 0.001 level

**Fig. 3:**
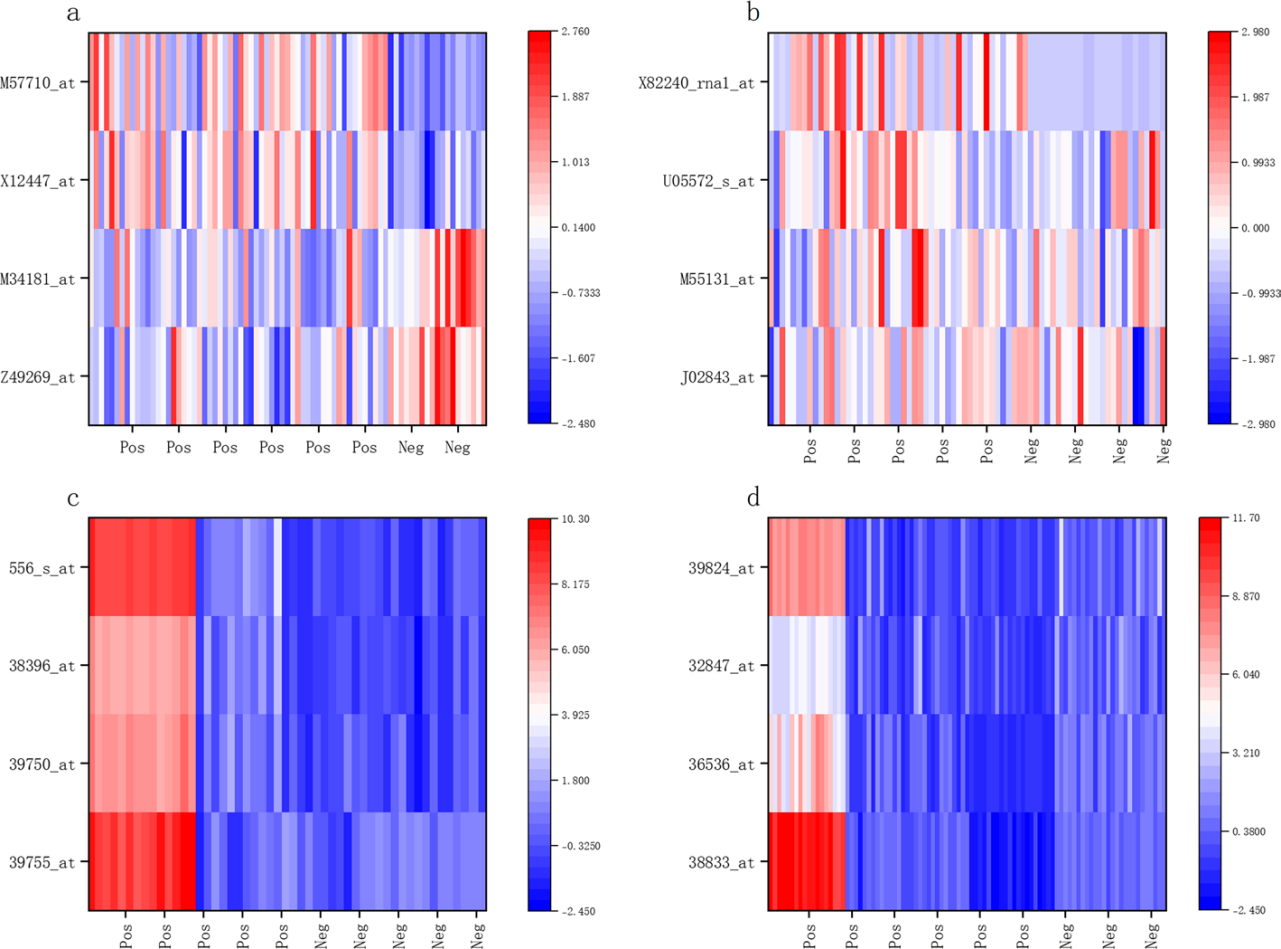
Heat map analysis of the features selected by the proposed method. **a** represents DLBCL data set, **b** represents leukemia data set, **c** represents prostate data set, and **d** represents ALL_4 data set

The heat map of biomarkers corresponding to probes in different sample intervals is shown in Fig. 3. In the process of heat map plotting, we performed Z-score normalization for each dataset separately, and then performed heat map plotting. The results in Fig. 3 show that the probes corresponding to these biomarkers are highly discriminated on different sample intervals, especially on the Prostate and All_4 datasets, which can significantly distinguish the samples in different intervals and can indicate that these biomarkers are significant.

To demonstrate the biological significance of the features selected by the proposed method, in the DLBCL dataset, we analyzed the number of literatures related to the disease reported on PubMed for the features selected by the proposed method, as the basis for judging the biological significance of the selected features. Table 5 shows the gene IDs corresponding to the four most important features selected by the proposed method on the DLBCL dataset, and the number of results returned when searching with the gene and the disease name as keywords. From the results, it can be seen that the features selected by the proposed method are all reported to be associated with the disease by different numbers of literatures, proving that the features selected by the proposed method are biologically meaningful.

**Table 5:** The features selected by the proposed method in the DLBCL data correspond to gene IDs and PubMed search analysis results, where PubMed Hits represents the number of search results when the keyword DLBCL and the corresponding gene ID are used together as search keywords

Prob ID	Gene	PubMed hits
Z49269_at	CCL14	7
M34181_at	PRKACB	18
X12447_at	ALDOA	15
M57710_at	LGALS3	149

**Fig. 4:**
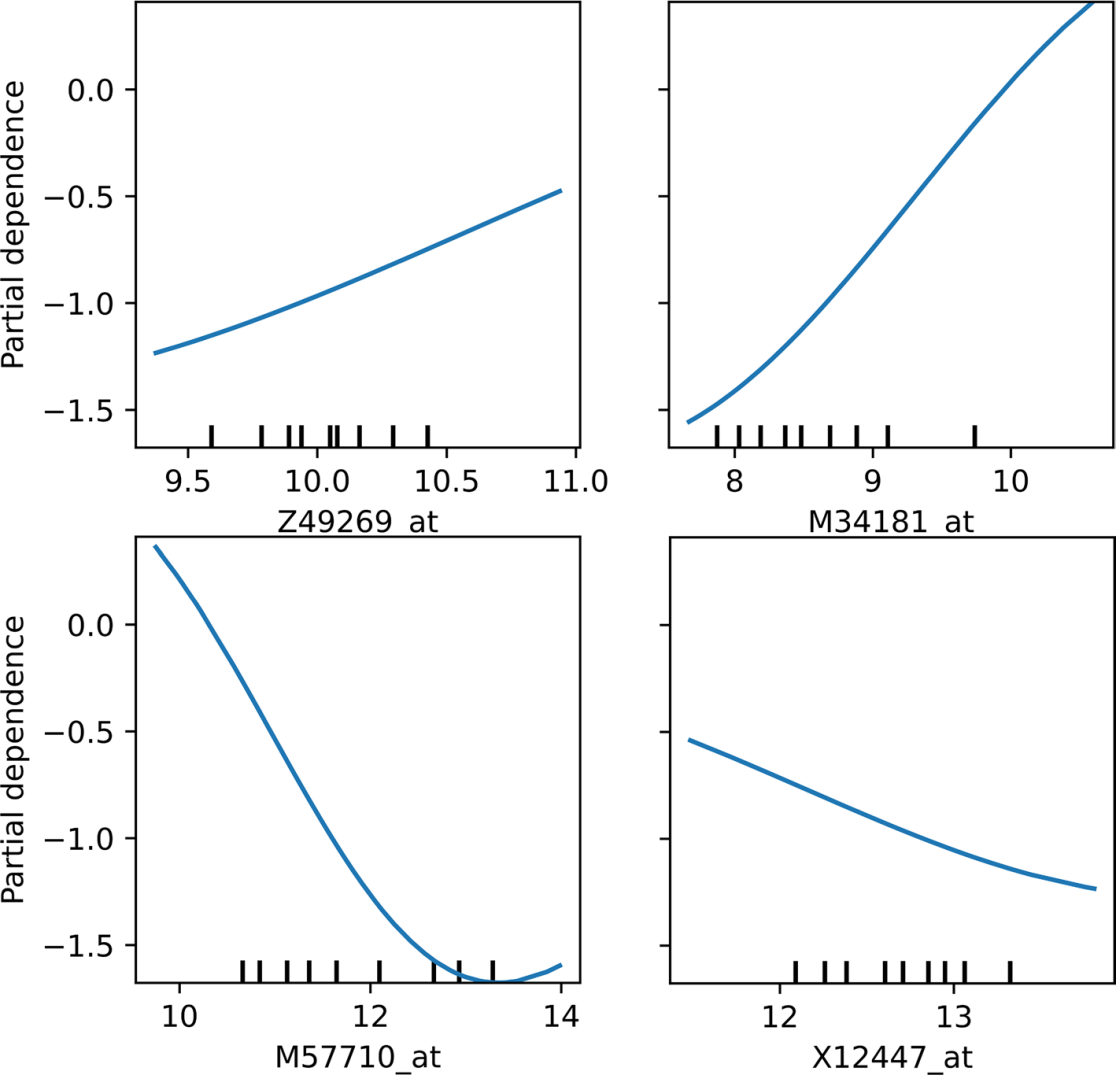
Partial dependency graph of the features selected by the proposed method on the DLBCL dataset

Furthermore, to demonstrate that the selected features are meaningful, we draw partial dependency graphs for the four selected features in the DLBCL data. The partial dependence graph can reflect the contribution of a feature to the model. Generally speaking, the slope of the important feature changes greatly, and the response curve changes sharply. The slope of the unwanted feature tends to be zero, and the response curve is close to a smooth straight line. Fig. 4 shows the partial dependency graph of the four features under SVM as the classification model. It can be seen that the four features selected by the proposed method have important contributions to the model, especially M34181_at and M57710_at, the response curves of these two features change drastically, has a more prominent contribution to the classification model.

## Discussion

In the results of Fig. 1, we analyze the changes in Acc and Auc metrics corresponding to the proposed method when different numbers of features are selected. From the results, we can see that the increase in the number of features in the feature selection task for microarray data is not effective in improving the classification accuracy of the model, and the inclusion of too many features may lead to a decrease in the classification accuracy of the model due to the introduction of redundant features. This illustrates the importance of the feature selection task for building disease classification models for microarray data and that too many features can increase the cost of clinical validation and testing.

The results of Table 2 show that the proposed method can effectively outperform the traditional feature selection methods by achieving higher classification accuracy with a smaller number of features due to the feature dependencies for feature selection. This proves the correctness and foresight of introducing actual feature dependencies and using graphical neural networks for the analysis direction. Furthermore, in Table 3, we compare the proposed method with some advanced hybrid feature selection methods, and the same can prove the advancedness of the proposed method. Therefore we believe that it is essential to introduce real feature dependencies for feature selection. Currently, the proposed method does not apply all the feature dependencies provided by GeneMANIA. We believe future research can further explore these feature dependencies to achieve more accurate and effective feature selection.

In Figs. 2 and 3, and the results in Table 4, we analyze the biological significance of the features selected by the proposed method. Unlike the current mainstream classical feature selection methods and hybrid feature selection methods, the proposed method does not entirely rely on classification accuracy as the fitness function for feature selection. However, it introduces actual feature dependencies, and we believe that introducing such dependencies can make the features selected by the proposed method more biologically meaningful. The experimental results also prove this point. The features selected by the proposed method are very significant in *p*-value, positive and negative sample distribution, and heat map, which can effectively distinguish between positive and negative samples.

In the experimental results in Table 5, we further demonstrate the biological significance of the features selected by the proposed method by analyzing the literature in the DLBCL dataset. All the features selected by the proposed method have been reported to be associated with the disease, so we have reason to believe that the feature selection method with the introduction of feature dependence can effectively select features with real biological significance. Moreover, the results in Fig. 4 also demonstrate that these features not only have true biological significance but are equally significant and contribute to the performance of the classification model.

Therefore, we believe that feature selection based on the introduction of real feature dependencies and analysis using advanced graphical neural networks can have the potential to surpass traditional feature selection methods and popular hybrid feature methods. In our future work, we will aim to fully exploit feature dependencies and adopt an analytical model for feature selection that is more in line with the characteristics of microarray data. We believe this work has important implications for biomarker selection.

## Conclusion

This paper proposes a biomarker selection algorithm based on a graph neural network. This method effectively uses the dependence between features and integrates a priori knowledge to select features together. The algorithm removes redundant features by clusting and uses eight feature evaluators to achieve accurate and efficient feature selection. The results show that the integration and prediction of the natural interaction between genes can effectively improve the accuracy and interpretability of the results. In addition, we also analyze the relationship between the number of features and classification accuracy and prove the effectiveness and reliability of the features selected by the proposed method.

## Method

### Dataset

Four DNA microarray datasets were used in this paper, namely DLBCL, Leukemia, Prostate and ALL_4, the details of these datasets are shown in Table 6. DLBCL contains 77 samples, of which there are 58 positive samples and 18 negative samples, the imbalance ratio is 3.05, and each sample contains 7129 features. Leukemia contains 72 samples, of which there are 47 positive samples and 25 negative samples, the imbalance ratio is 1.88, and each sample contains 7129 features. Prostate contains 102 samples, of which there are 52 positive samples and 50 negative samples, the imbalance ratio is 1.04, and each sample contains 12625 features. ALL_4 contains 93 samples, of which there are 26 positive samples and 67 negative samples, the imbalance ratio is 0.38, and each sample contains 12625 features. Datasets and GPL files can be downloaded from https://github.com/xwdshiwo/BioFSDatasets.

**Table 6:** The dataset used in this paper, Ur means Unbalance rate

Dataset	Samples	Pos	Neg	Features	Ur
DLBCL	77	58	19	7129	3.05
Leukemia	72	47	25	7129	1.88
Prostate	102	52	50	12,625	1.04
ALL_4	93	26	67	12,625	0.38

### The proposed framework of our method

**Fig. 5:**
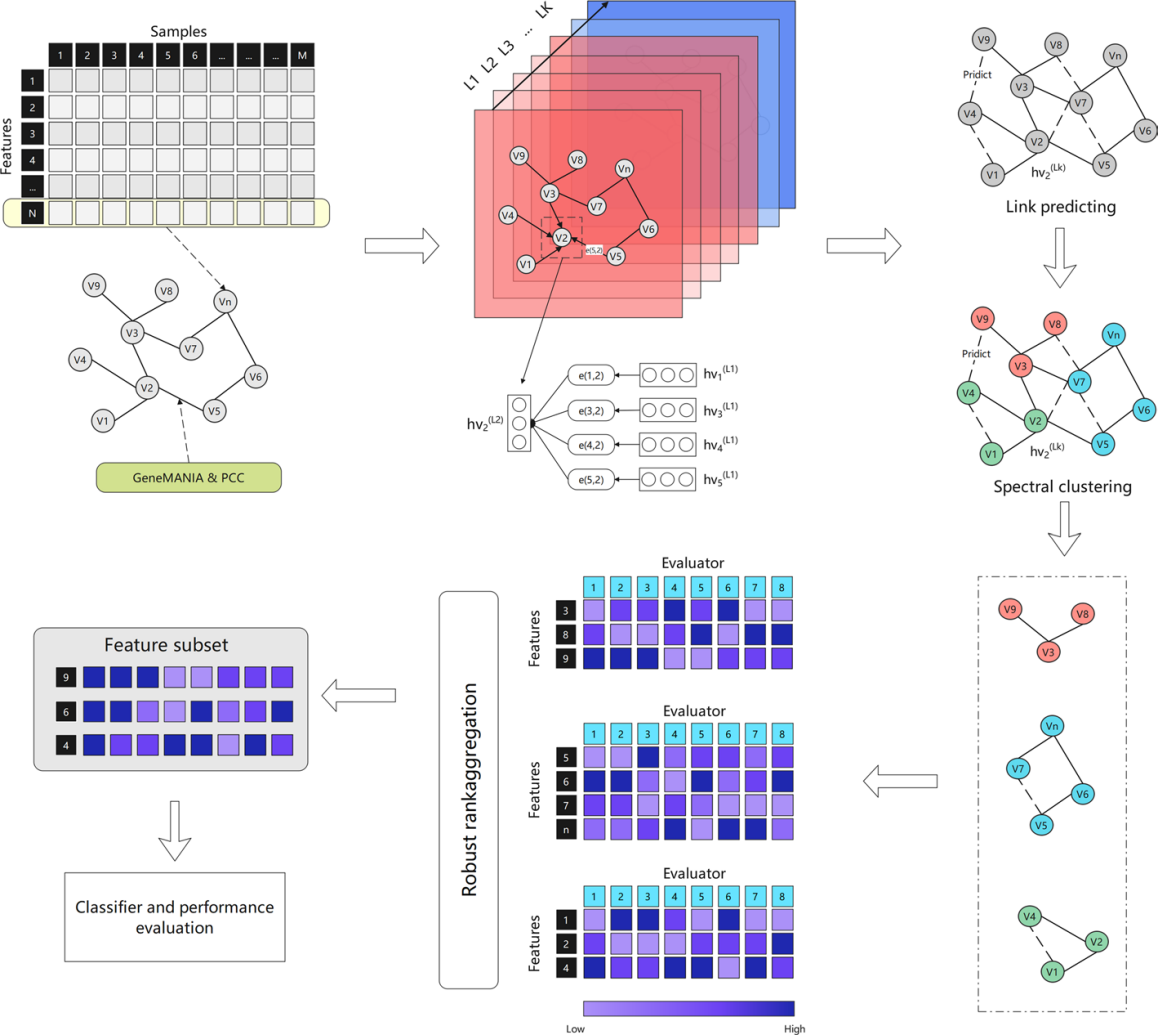
The overall framework of the proposed approach: The gene relationship data is obtained from GeneMANIA, the expression of each gene in positive and negative samples is embedded as node information, and the gene relationship data and Pearson correlation coefficient are embedded as edges after passing through a layer of softmax function. The graph neural networks’ information dissemination and aggregation process is carried out. The dependency relationship is predicted by the link prediction method, and spectral clustering is carried out to delete redundant features. The feature of each subgraph is evaluated, eight kinds of evaluators are used, the ranking information is aggregated by the robust ranking method, and the feature subset is finally output

The feature selection framework designed in this paper is shown in Fig. 5. The first step of the algorithm is to construct the graph structure. The characteristic information from the microarray data is used as the initial embedding representation of the node, and the physical interaction information from GeneMANIA and the Pearson correlation coefficient of the node are used as the edge information of the node after passing through a layer of softmax function. Then, we use the information propagation and aggregation function to embed the nodes to enrich the node information deeply. Then we construct positive samples by randomly deleting the head and tail links of known link nodes and construct negative samples by randomly adding some links. We realize the link prediction of the edge by training a loss function and cluster on the graph after link prediction to delete redundant features. In each clustering subgraph, eight feature evaluators are used to evaluate the feature weight, and the RRA method is used to sort the feature weight comprehensively. Finally, the final feature subset is generated, and the classification model is established to analyze further and evaluate the feature subset.

### Graph structure establishment

The sample-set in microarray data is defined as $S=\left\{ S_{1}, S_{2}, \ldots , S_{M}\right\}$, in which a n-dimensional feature vector $S_{i}=\left\{ F_{1}^{i}, F_{2}^{i}, \ldots , F_{N}^{i}\right\}$, represents each sample. Each feature is taken as node *v* in the graph. The physical correlation between two features *i* and *j* obtained from GeneMANIA is expressed as $e_{(v i, v j)}^{G m}$. The Pearson correlation coefficient between two nodes with physical correlation is expressed as $e_{(v i, v j)}^{P e}$. The correlation of the two nodes is calculated by the function shown in Eq. 1 and used as edge $e_{(v i, v j)}$.

1 $$\begin{aligned} e_{(v i, v j)}={\text {MEAN}}\left( e_{(v i, v j)}^{P e}+e_{(v i, v j)}^{G m}\right) \end{aligned}$$

Then we can get graph $G=\{V, E\}$, where *V* represents the set of all nodes and *E* represents the set of all edges. The initial embedding of each node is expressed as the eigenvector $h_{v i}^{0}=\left[ S_{1}^{F i}, S_{2}^{F i}, \ldots , S_{M}^{F i}\right]$ composed of the eigenvalues on each sample. In this way, we get the graph structure representation composed of the original microarray expression matrix and a priori knowledge information.

### Information propagation and aggregation

Information propagation is one of the essential components of a graph neural network. Its purpose is to make each node have the feature vector representing the global information to better carry out the following task. In the process of message propagation, firstly, as described in the structure establishment part of the figure, initialize the eigenvector $h_{v i}^{0}=\left[ S_{1}^{F i}, S_{2}^{F i}, \ldots , S_{M}^{F i}\right]$ of each node and define $N_{(v i)}$ to represent the first-order neighborhood of node *vi*. then the aggregation operation is shown in Eq. 2 to obtain the state vector of the next layer of the node.

2 $$\begin{aligned} h_{v i}^{K}=\sum _{v j \in N_{(v i)}}\left( h_{v j}^{K-1} * e_{(v i, v j)}\right) / {\text {Num}}\left( N_{(v i)}\right) \end{aligned}$$

where *K* represents the number of layers of the current graph neural network, and ${\text {Num}}(*)$ represents the number of first-order neighborhood nodes. We believe that when the difference of eigenvectors after two aggregations is less than the given threshold $\varepsilon$, the current graph reaches a stable state, the next layer of propagation will not be carried out.

In the information aggregation stage, each node splices the current layer’s state vector with the previous layer’s state vector and obtains the final state vector representation of the current layer through the nonlinear activation layer, as shown in Eq. 3.

3 $$\begin{aligned} h_{v i}^{K}=\sigma \left( C O U N C A T\left( h_{v i}^{K-1}, h_{v i}^{K}\right) \right) \end{aligned}$$

where $\sigma$ is the nonlinear activation function, representing the vector splicing operation. Then, the normalized representation of the node vector is carried out as shown in Eq. 4, and the *k*-th layer state vector of the node is updated.

4 $$\begin{aligned} h_{v_{i}}^{K} \leftarrow h_{v_{i}}^{K} /\left\| h_{v_{i}}^{K}\right\| _{2}, v_{i} \in v \end{aligned}$$

In the experiment, the above process is repeated until the difference between *k* layer and $k-1$ layer state vectors of all nodes is less than the given threshold $\varepsilon$, then stop the iteration and record the number of iteration layers *L*, and finally get the *L*-layer state vector of all nodes.

### Link prediction

The purpose of link prediction is to predict the hidden relationship between two nodes, taking advantage of feature correlation and node high-order connectivity to prepare for further analysis. Feature selection uses the hidden state information of the node for prediction. After the information dissemination and aggregation of the graph neural network, the node has a state vector representing the global information, which can better carry out the prediction task.

In the process of link prediction, we first need to build positive and negative samples. Taking node *vi* as an example, we break any head and tail links connected to node *vi* in graph *G*, and randomly take *vi* as the central node to sample several new edges $e_{new}$. if $e_{n e w} \in E$, it will be marked as positive samples, otherwise it will be marked as negative samples. Then we record the similarity between vi as the central node and all the nodes connected to the new edge (taking node *vr* as an example, it is a node connected to node *vi* through $e_{new}$). The calculation method is shown in Eq. 5.

5 $$\begin{aligned} \begin{aligned} {\text {sim}}\left( v_{j}, v_{r}\right) =\frac{\sum _{\varphi =1}^{\pi } z_{v_{j}}^{\varphi } \times \sum _{\varphi =1}^{\pi } z_{v_{r}}^{\varphi }}{\sqrt{\sum _{\varphi =1}^{w}\left( z_{v_{j}}^{\varphi }\right) ^{2}} \times \sqrt{\sum _{\varphi =1}^{w}\left( z_{v_{r}}^{\varphi }\right) ^{2}}} \end{aligned} \end{aligned}$$

where $z_{v_{j}}^{\varphi }$ represents the value of eigenvector $v_{j}$, and *w* represents the eigenvector dimension. We set the positive sample set as *Pos* and the negative sample set as *Neg* and establish the loss function as shown in Eq. 6.

6 $$\begin{aligned} L=MEAN_{\left( v_{j}, v_{r}\right) \in {\text {Pos}}}\left[ -\log \left( \sigma \left( {\text {sim}}\left( v_{j}, v_{r}\right) \right) \right) -\sum _{\left( \overline{v_{j}}, \overline{v_{r}}\right) \in \mathrm {Neg}} \log \left( \sigma \left( {\text {sim}}\left( \overline{v_{j}}, \overline{v_{r}}\right) \right) \right) \right] \end{aligned}$$

where *L* represents the loss value of the loss function, $\left( v_{j}, v_{r}\right) \in {\text {Pos}}$ represents the edge of any group of positive data samples,$\left( {\bar{v}}_{j}, {\bar{v}}_{r}\right) \in \mathrm {Neg}$ represents the edge of any group of positive data samples, $\sigma$ is a nonlinear activation function. The random gradient descent algorithm is used to train the model, and the loss value *L* in training is retained. When the difference between the loss values of the two training is less than $\varepsilon$, the training is stopped. At the same time, we calculate the mean predictive rank (*MRR*) of each prediction graph in training. The calculation method is shown in Eq. 7, and select the optimal graph as the final result according to the *MRR*. In this way, we get graph $G^*$, which has more prosperous relational attributes than graph *G*.

7 $$\begin{aligned} M R R=\frac{1}{\varepsilon } \sum _{\tau =1}^{\varepsilon } \frac{1}{{\text {rank}}_{\tau }} \quad \tau =1,2, \ldots , \varepsilon \end{aligned}$$

*MRR* represents the average reciprocal rank, and rank represents the rank number of the scores from highest to lowest when the $\tau$-th edge in the positive sample set scores the corresponding $\varepsilon$-th edge in the negative sample set.

### Spectral clustering and feature ranking

After obtaining the new gene relationship graph $G^*$, we can use its prosperous gene relationship to cluster redundant features and find the feature gene with the most abundant information in each sub-cluster. We use spectral clustering to cluster features. The idea of spectral clustering comes from our previous research [31]. The process is as follows:

Define all nodes in the new gene graph $G^*$ as *E*, that is, $E=\left( e_{1}, e_{2}, \ldots , e_{\zeta }\right)$, $\zeta$ represents the total number of nodes in the gene graph $G^*$. Use Eq. 8 to calculate the similarity between any two nodes $(v_i,v_j)$, and $w_(v_i,v_j)$ will form an $\zeta$ dimensional similarity matrix *W*.

8 $$\begin{aligned} w_{v i, v j}=\sum _{v i=1, v j=1}^{\zeta } \exp \frac{-\left\| e_{v i}-e_{v j}\right\| ^{2}}{2 \Omega ^{2}}, \quad e_{v i}, e_{v j} \in E \end{aligned}$$

$\Omega$ uses to control the neighborhood width of nodes. Calculate the sum of all elements in each row of the similarity matrix *w* to obtain $\left\{ d_{1}, d_{2}, \ldots , d_{\eta }, \ldots d_{\zeta }\right\}$, where $d_{\zeta }$ represents the sum of all elements in the row, and use $\left\{ d_{1}, d_{2}, \ldots , d_{\eta }, \ldots d_{\zeta }\right\}$ to construct the diagonal matrix with *D* dimension, then use Eq. 9 to calculate laplacian matrix $L_{\text{ reym } }$.

9 $$\begin{aligned} L_{\text{ reym } }=D^{\frac{-1}{2}}(D-W) D^{\frac{-1}{2}} \end{aligned}$$

Calculate the eigenvalues of the Laplace matrix $L_{\text{ reym } }$, and sort the eigenvalues in the order from small to large. According to the number $\mu$ of clustering clusters, take the first $\mu$ eigenvalues and calculate the corresponding eigenvector $\left\{ \chi _{1}, \chi _{2}, \ldots , \chi _{\mu }\right\}$. use the $\mu$ eigenvectors $\left\{ \chi _{1}, \chi _{2}, \ldots , \chi _{\mu }\right\}$ to form the matrix *U* of rows and columns, that is, $U=\left\{ \chi _{1}, \chi _{2}, \ldots , \chi _{\mu }\right\}$.

The K-means clustering algorithm is used to cluster the eigenvectors in each row of matrix *U* to obtain $\left\{ C_{1}, C_{2}, \ldots , C_{v}, \ldots , C_{\mu }\right\}$, where $C_v$ represents the cluster clustered by the eigenvectors in row V. According to the obtained cluster $\left\{ C_{1}, C_{2}, \ldots , C_{v}, \ldots , C_{\mu }\right\}$, all nodes in the new gene relationship graph $G*$ are divided into $\mu$ groups to obtain $\mu$ subgraph, which is recorded as Eq. 10.

10 $$\begin{aligned} G^{*}=\left[ G_{1}, G_{2}, \ldots , G_{v}, \ldots , G_{\mu }\right] =\left[ \left( v_{1}^{\prime }, \varepsilon _{1}^{\prime }\right) ,\left( v_{2}^{\prime }, \varepsilon _{2}^{\prime }\right) , \ldots ,\left( v_{v}^{\prime }, \varepsilon _{v}^{\prime }\right) , \ldots ,\left( v_{\mu }^{\prime }, \varepsilon _{\mu }^{\prime }\right) \right] \end{aligned}$$

where $G_v$ represents the *v* subgraph, the *v* subgraph represents $\left( v_{v}^{\prime }, \varepsilon _{v}^{\prime }\right)$, $v_{v}^{\prime }$ represents all node sets in the subgraph $G_v$, and $\varepsilon _{\mu }^{\prime }$ represents all edges in the subgraph $G_v$.

In the graph $G^{*}=[G_{1}, G_{2}, \ldots , G_{v}, \ldots , G_{\mu }]$, each subgraph includes several nodes, and since these nodes are obtained after information propagation and aggregation by graph neural networks and spectral clustering, the features corresponding to these nodes can be considered as highly redundant. In order to select a subset of features with low redundancy, we use eight different feature evaluation methods for feature evaluation and ranking in each subgraph $G_{1}, G_{2}, \ldots , G_{v}, \ldots , G_{\mu }$, which include L1 regularization, L2 regularization, t-test, correlation coefficient evaluation, decision tree, random forest, stability selection, and linear regression. Each feature evaluation method evaluates the features corresponding to each subgraph $G_{v}$ separately and generates a feature ranking list $ra_v={ra1,ra2,ra3, \ldots ,ra8}$, and then we use the Robust Rank Aggregation (RRA) method to fuse these ranking lists and finally generate a uniform ranking list corresponding to graph $G_{v}$, and take the best ranked feature as the output of this subgraph. RRA is a widely used feature ranking fusion method, which can synthesize the results of multiple evaluators and output the best feature subset. After performing this operation for each subgraph, we finally obtain a subset of features after feature selection on the whole dataset. Usually, the clusters can be selected according to downstream tasks or determined according to elbow rules. In the Results part, we analyzed the number of *K*.

## Data Availability

The public data set used in our experiment is from the GEO (Gene Expression Omnibus) database, which can be obtained through the following website: https://www.ncbi.nlm.nih.gov/geo. https://github.com/xwdshiwo/BioFSDatasets

## Abbreviations

GNN: Graph neural network
RFE: Recursive feature elimination
GA: Genetic algorithm
KNN: K-nearest neighbor
PSO: Particle swarm optimization
DT: Decision tree
RF: Random forest
LR: Lasso regression
MIN: Mutual information maximization
SGA: Standard genetic algorithm
CFS: Correlation-based feature selection
MB: Markov blank
